# The origin of the criticality in meme popularity distribution on complex networks

**DOI:** 10.1038/srep23484

**Published:** 2016-03-24

**Authors:** Yup Kim, Seokjong Park, Soon-Hyung Yook

**Affiliations:** 1 Department of Physics and Research Institute for Basic Sciences, Kyung Hee University, Seoul 130-701, Korea

## Abstract

Previous studies showed that the meme popularity distribution is described by a heavy-tailed distribution or a power-law, which is a characteristic feature of the criticality. Here, we study the origin of the criticality on non-growing and growing networks based on the competition induced criticality model. From the direct Mote Carlo simulations and the exact mapping into the position dependent biased random walk (PDBRW), we find that the meme popularity distribution satisfies a very robust power- law with exponent *α* = 3/2 if there is an innovation process. On the other hand, if there is no innovation, then we find that the meme popularity distribution is bounded and highly skewed for early transient time periods, while it satisfies a power-law with exponent *α* ≠ 3/2 for intermediate time periods. The exact mapping into PDBRW clearly shows that the balance between the creation of new memes by the innovation process and the extinction of old memes is the key factor for the criticality. We confirm that the balance for the criticality sustains for relatively small innovation rate. Therefore, the innovation processes with significantly influential memes should be the simple and fundamental processes which cause the critical distribution of the meme popularity in real social networks.

Complex networks are ubiquitous and play important roles in many systems associated with human activities^1^,^2^. One of the well-known examples is the social network service (SNS) through the Internet, which permeates our everyday life with significant influence. Human activities in such online environment explosively increase and show novel properties of collective behavior. Recently, the available data obtained from various SNSs have enabled to study the various human and social phenomena on a large scale^3^,^4^,^5^,^6^,^7^. Especially, the spreading of cultural entities such as news, technology, word, and idea through the online society shows very interesting phenomena. During the spreading of such cultural entities, they evolve via replication and mutation in human culture^8^. This resembles the evolution of gene in biological environments. Such dynamical cultural entities are called as “meme”. The term “meme” was first coined by Dawkins as a cultural analogy with gene^9^. The replication and mutation inevitably cause competitions among memes. In human culture some entities, among the many roughly equivalent ones, quickly become extremely popular, while others are rarely chosen and disappear. This implies that the meme evolves under a given social influence and competitions. Examples include baby names^3^, apps on SNS^4^,^5^, retweeted messages or forwarded message on SNS^6^,^10^, or video views on YouTube^7^.

Interestingly, in these examples the meme popularity distribution, *P*(*n*), which is defined as the probability that a randomly chosen meme has been selected *n* times for being forwarded to the connected neighbors, scales approximately as a power-law^4^,^5^,^10^


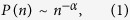


which is the characteristic feature of the criticality. The exponent *α* in those examples is known to be less than two, and typically close to 1.5, which is notably different from the Yule-Simon process^11^,^12^ or preferential attachment^13^ in which *α* ≥ 2, but similar to the exponent for the avalanche distribution in self-organized criticality (SOC)^14^. This finding is remarkable. However, the origin of the criticality in meme popularity is not yet clearly understood even though there have been a few attempts to explain the observed power-law in meme popularity distribution^10^,^15^. In this Report, to find the origin of the criticality we use simple stochastic models without any assumption about the detailed properties of memes.

## Previous Studies and Problem Statement

Recently, Weng *et al.* proposed a simplified model for the spreading of memes through SNS^10^. In this model, they assumed a frozen network with the fixed number of agents. From the numerical simulations and comparison with real data, they showed that the heavy-tailed distribution of meme popularity and life time can be obtained by the limited attention of each agent and the competition among memes. Inspired by ref. 10 and the value of *α* which is very close to that of the avalanche distribution in SOC, Gleeson *et al.* proposed a competition induced criticality (CIC) model on frozen networks^15^. In CIC model if there are only two memes, then the time evolution of the meme population can be described by the rate equations which are similar to that for the standard susceptible-infected model in epidemic spreading as addressed in ref. 15. However, if the number of memes are not bounded, then the model cannot be simply mapped into the standard epidemic spreading model which has finite number of states. The CIC model is simpler than that of Weng *et al.* and analytically tractable by a mapping to the branching process. When the outgoing-degree distribution, *p*_*out*_(*k*), has finite second moment, they derived


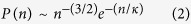


in the limit that both *n* and the age of a meme go to infinity. From this analytic result, they argued that *P*(*n*) ~ *n*^−(3/2)^ or *α* = 3/2 for *n* < κ^15^. When *p*_*out*_(*k*) ~ *k*^−*y*^ with 2 < *γ* < 3, they also argued to derive that *α* = *γ*/(*γ* − 1) if the probability to generate a new meme is zero and *α* = *γ* otherwise^15^.

Even though the heavy-tailed distribution of *P*(*n*) and the value of *α* can be described by those models under a certain kind of conditions^4^,^5^,^10^, the suggested models are still far from describing meme propagation on real world networks properly. In the real world, size of SNS grows exponentially^16^. Thus, the effect of growth must be one of the essential factors to understand the properties of real social networks. One of well-known example is the topological property of network. For example, it is analytically and numerically proved that the degree distribution, *p*(*k*), for non-growing network does not follow a power-law, even though new links are connected to the old nodes with a preferential attachment rule or by the Yule-Simon process^17^. This clearly shows that the growth process is very crucial to determine the topological properties of network itself. Thus, finding the effect of a growing network on the meme popularity is very important to understand various dynamical properties of meme on many real social networks. In order to study the effect of network growth on the meme popularity, we propose a new CIC model on growing networks (CICGN) in this Report. The essential feature of the model is that a newly attached node generates a new meme. This model is shown to be exactly mapped into a position-dependent biased random walk (PDBRW), in which the hopping probability depends on the location of the walker. Biased random walk itself has many theoretical importance in diverse disciplines^18^. Using the numerical analyses of CIC and CICGN models and the exact mapping into PDBRW, we find that the balance between the creation of new memes by the innovation process and the extinction of old memes on the growing networks is the key factor for the criticality with *α* = 3/2. We confirm that the balance for the criticality sustains for relatively small innovation rate in CICGN model. In real social networks, the innovations with impactable memes should not happen very often. Therefore, the innovation processes with significantly influential memes should be the simple and fundamental processes which cause the power-law or heavy-tailed distribution of the meme popularity in various real social networks.

## Results

### Model

The CICGN is similar to the CIC model suggested by Gleeson *et al.*^15^, but we incorporate the growth with innovation process in the model. In this model each node (or agent) has a screen. The screen has a meme of the current interest of the node. Each screen has capacity for only one meme. At *t* = 0, we start from a network of *N(t* = *0)* = *N*_*0*_ nodes and *T(t* = *0)* = *T*_*0*_ memes are randomly distributed to the nodes. Resultantly, the number of nodes with a same meme is *N*_0_/*T*_0_. At each time step (with time increase Δ*t* = 1/*N*(*t*)), *growth with innovation* is taken with probability *μ* or *propagation process* is taken with probability 1 − *μ*. In *the growth with innovation*, a new node with a new meme is generated and attached to some of nodes on the network. Thus, both *N*(*t*) and *T*(*t*) increase by one. Furthermore, the new meme of the new node is propagated to the connected neighbors. In *the propagation process*, a randomly selected node on the network propagates its meme to all its connected neighbors. When a meme of type *u* is propagated, the popularity of meme of type *u* at *t*, *n*_*u*_(*t*), increases by 1.

In order to test the effect of innovation itself, we also reinvestigate the CIC model with innovations of new memes on non-growing networks^15^. At each time step in the CIC model, a randomly chosen node innovates a new meme and propagates it to all its neighbors (innovation) with probability *μ*′ or propagates the current meme to all its connected neighbors with 1 − *μ*′ (propagation).

### Growing Network

In order to study the effect of underlying topologies we consider two types of networks, random networks (RN) and scale-free networks (SFN). Although in ref. 15 directed networks are used to represent underlying topologies for social networks, we check that the obtained popularity distribution behaves in the same way on undirected networks. Thus, in the following simulations we use undirected networks to represent topologies for synthetic social networks. In CICGN model at each time step a new node is added with probability *μ* and linked to the existing nodes. To generate a growing RN with a fixed mean degree, 〈*k*〉, we attach the new node to all existing nodes with probability 〈*k*〉/*N*(*t*). To generate a growing SFN, we use the preferential attachment model with attractiveness^19^. In this model a new node is added with *m* edges that connect the new node to *m* different nodes already present in the network. When we choose nodes to connect to the new node, a node *i* with degree *k*_*i*_ is selected among *N*(*t*) old nodes with probability


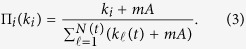


Here, *A* is the attractiveness of node *i* which is set to be the same for all nodes. The degree distribution of the resulting growing SFN is known to satisfy the power-law *p*(*k*) ~ *k*^*−γ*^ with *γ* = 3 + *A* (*A* < 0)^19^.

### Mapping to a position-dependent biased random walk

#### Non-growing network without innovation: *μ* = 0 in CICGN model or *μ*′ = 0 in CIC model

**Figure 1:**
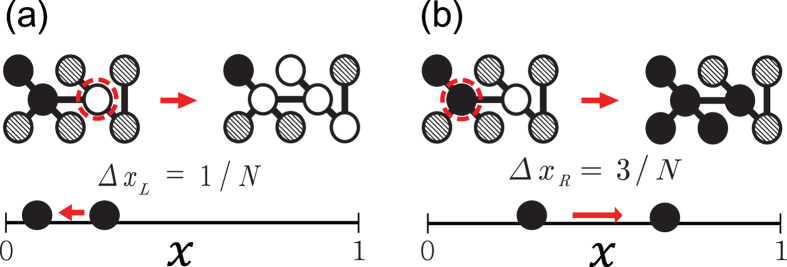
Schematic diagram for the mapping from propagation of memes on a network to PDBRW on 1-dimensional space. Different filled patterns of nodes denote the different memes. Let *M*(*t*) be the number of the memes denoted by black circles. (**a**) The propagation process in which a black meme disappears by the propagation of the selected white meme (top panel) is mapped to the process of *M* → *M* − 1 or the hopping to the left by Δ*x*_*L*_ = 1/*N* in PDBRW(bottom panel). (**b**) The propagation process in which the selected black meme propagates to three connected neighbors(top panel) is mapped to the process of *M* → *M* + 3 or the hopping to the right by Δ*x*_*R*_ = 3/*N* in PDBRW (bottom panel).

The dynamical properties of the meme can be exactly mapped into the one-dimensional PDBRW. Let us first consider the case of *μ* = 0 in CICGN model or 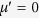 in CIC model, in which the underlying network does not grow and there is no innovation of new memes. Let *M*(*t*) be the number of a certain meme *i* at *t* and *x*(*t*) be the fraction of nodes with the *i* meme, i.e., *x*(*t*) = *M*(*t*)/*N*. If we interpret *x*(*t*) as the location of a walker at *t* in one-dimensional space, then the walker moves within the interval [0, 1] (see the bottom panel of Fig. 1). During each time step Δ*t*, if a different meme *j*(≠*i*) is selected and propagated to the nodes with the *i* meme as in Fig. 1(a), then *M*(*t*) decreases because the *i* memes can be overwritten by the *j* meme. On the other hand, if an *i* meme is selected and propagated to the nodes having a different meme, then *M*(*t*) increases (see Fig. 1(b)). The increase (decrease) of *M*(*t*) moves the walker to the right (left). Therefore, the probability that the walker hops to the right corresponds to the probability to select a node with the *i* meme. Thus, the probability to move right becomes *P*_*R*_(*t*) = *x*(*t*). Similarly, the probability to move left is given by *P*_*L*_(*t*) = 1 − *x*(*t*). When the walker moves to the right (left), the average distance Δ*x*_*R*_(*t*) (Δ*x*_*L*_(*t*)) to move to the right (left) is Δ*x*_*R*_(*t*) = *zx*(*t*)(1 − *x*(*t*)) (Δ*x*_*L*_(*t*) = *z*′(1 − *x*(*t*))*x*(*t*)). Here, *z* (*z*′) is the fraction of nodes connected to the selected one with the *i* (*j*) meme. On a complex network with the degree distribution *p*(*k*), we randomly select a degree, *k*, from *p*(*k*) and set *z* = *k*/*N* (*z*′ = *k*′/*N*) to implement the degree heterogeneity. The popularity of a certain meme, *n*, can be easily obtained by counting the total number of hopping to the right in PDBRW.

#### Non-growing network with innovation: *μ*′ > 0 in CIC model

When *μ*′ > 0 in CIC model, the mapping to the PDBRW is a little sophisticated due to the increase of memes through the innovation process, while the size of network remains unchanged. In the innovation process at *t*, with the probability *μ*′*x*(*t*) a new meme is generated to replace one of *i* memes and 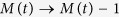. Simultaneously, the newly generated meme is propagated to the connected neighbors. Thus, the innovation process makes the walker move to the left by 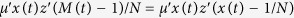. With the probability *μ*′(1 − *x*(*t*)), the generated meme overwrites a meme other than *i* and the accompanying propagation moves the walker to the left by *z*′*x*(*t*). Thus, the innovation process makes the walker move to the left by


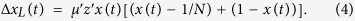


With probability 1 − *μ*′ the same processes as CICGN with *μ* = 0 occur. Thus,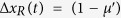 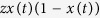 and 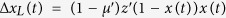.

#### Growing network: *μ* > 0 in CICGN model

In PDBRW model with *μ* > 0, the effect of the network growth should be considered in the following way. If a new node with a new meme is created with probability *μ* and attached to some nodes on the network, then the total number of nodes increases by one, i.e., 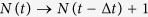, while *M*(*t*) remains unchanged because the new meme should not be the type *i*. Simultaneously, the new meme is propagated to the connected neighbors, which moves the walker to the left by


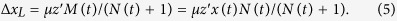


If 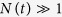, Eq. (5) becomes the same form as Eq. (4). This result means that the innovation process should be the same on the infinite-sized networks whether the network grows or not. In contrast, with probability 1 − *μ* the same processes as CICGN with *μ* = 0 occur. Thus, 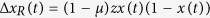 and 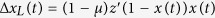.

### Numerical results

#### CICGN model with *μ* = 0 and small *T*_0_

**Figure 2:**
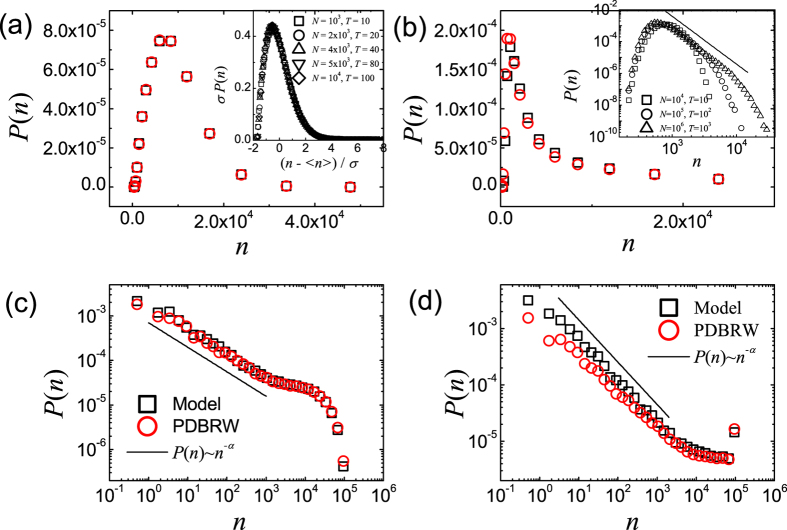
Plots of *P*(*n*) of the CICGN model with *μ* = 0, *T*_0_ = 10 and *N*_0_ = 10^4^, for *t* = 0 ~ 10 on RNs (**a**), for *t* = 0 ~ 10 on SFNs with *γ* = 2.5 (**b**), for *t* = 40 ~ 50 on RNs (**c**) and for *t* = 40 ~ 50 on SFNs with *γ* = 2.5 (**d**). (Black) Squares are *P*(*n*) obtained from direct Monte Carlo simulation of the CICGN model and (red) circles are those from the PDBRW. The solid lines represent the relations *P*(*n*) ~ *n*^−*α*^ with *α* = 0.6 ± 0.1 (**c**) and *α* = 0.8 ± 0.1 (**d**). Insets: (**a**) Scaling plot of *σP*(*n*) against 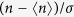 for various *N*_0_ with *T*_0_/*N*_0_ = 10^−3^ on RN 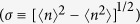. (**b**) Plots of *P*(*n*) for various *N*_0_ with *T*_0_/*N*_0_ = 10^−3^ on SFN against *n*. The solid line represents the relation *P*(*n*) ~ *n*^−*α*^ with *α* = 3.

When *μ* = 0 the underlying network does not grow and there is no innovation process. Thus, the dynamic rules for the propagation of meme in CICGN model become the same as those in CIC model with *μ*′ = 0^15^. *P*(*n*) in CIC model was argued to satisfy the power-law with *α* = 3/2 in the limits *t* → ∞ and *n* → ∞ when the second moment of *p*(*k*) is finite and *μ*′ = 0. They also presented the numerical results of *P*(*n*) in CIC model with *μ*′ = 0, and suggested that *α* = 3/2 even for small *n* and *t*. However, as we show in Fig. 2, *P*(*n*) strongly depends on *t* and the ratio *T*_0_/*N*_0_ in contrast to the results in ref. 15. As shown in Fig. 2(a,b), when *T*_0_/*N*_0_ < 10^−2^, we find that the measured *P*(*n*) both on RN and SFN leads to a single peaked and skewed distribution for *t* = 0 ~ 10. When *T*_0_/*N*_0_ is small enough, for example *T*_0_/*N*_0_ < 10^−4^, *P*(*n*) on RN is almost symmetric (which is not shown), and very well approximated by the Gaussian distribution. Skewness of *P*(*n*) increases with *T*_0_/*N*_0_ as shown in Fig. 2(a,b). Even though the skewness is relatively large, we find that *P*(*n*) on RN is well described by


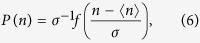


as shown in the inset of Fig. 2(a). Here *f*(*x*) is a universal function and 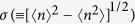 scales as *σ* ~ (*T*_0_/*N*_0_)^1/2^ like the Gaussian distribution. On the other hand, *P*(*n*) on SFN can not be described by a single universal function such as Eq. (6) . The data in the inset of Fig. 2(b) shows that *P*(*n*) for *N*_0_ = 10^4^ and *T*_0_ = 10 can be well approximated by the lognormal distribution. However, as we increase *N*_0_ with fixed ratio *T*_0_/*N*_0_(=10^−3^), *P*(*n*) becomes highly skewed and *P*(*n*) for large *n*(>10^3^) can be approximated by Eq. (1) with *α* = 3. The obtained value of *α* for *P*(*n*) on SFN with large *N*_0_ is quite close to value of *α* obtained from the Yule-Simon process^11^,^13^.

As *t* increases, some memes are more selected than others and sometimes memes can disappear due to competition among them. And, finally, there is only one meme left in the system as *t* → ∞. Interestingly, *P*(*n*) for intermediate time interval is well described by the power-law or heavy-tailed distribution as shown in Fig. 2(c,d). From the best fit of the data obtained from the numerical simulations of the model to Eq. (1) we obtain *α* = 0.6 ± 0.1 on RNs and *α* = 0.8 ± 0.1 on SFNs. The numerical results for the PDBRW are also displayed in Fig. 2, which show good agreements with those from the direct Monte Carlo simulation of the CICGN model.

#### CICGN model with *μ* = 0 and large *T*_0_

**Figure 3:**
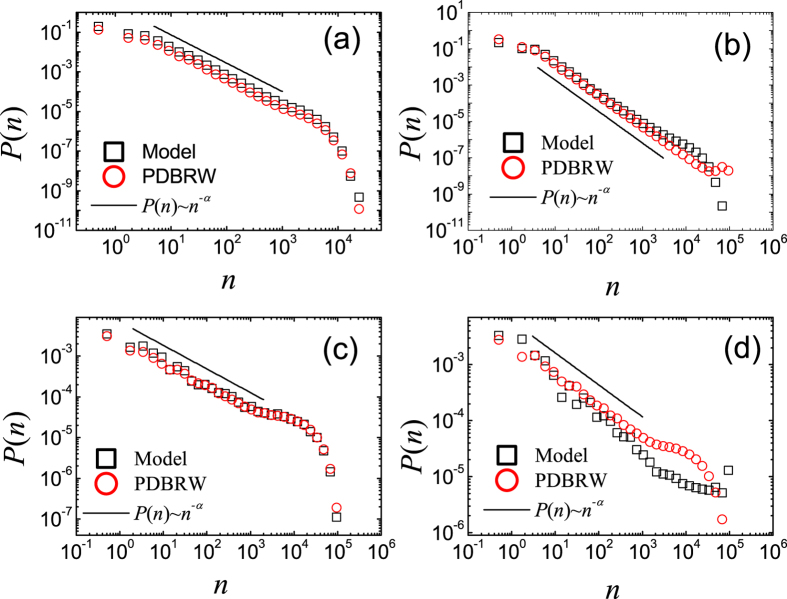
Plots of *P*(*n*) of the CICGN model with *μ* = 0, *T*_*0*_ = 10^3^ and *N*_0_ = 10^4^ for *t* = 0 ~ 10 on RNs (**a**), for *t* = 0 ~ 10 on SFNs with *γ* = 2.5 (**b**), for *t* = 40 ~ 50 on RNs (**c**) and for *t* = 40 ~ 50 on SFNs with *γ* = 2.5 (**d**). (Black) Squares are *P*(*n*) obtained from direct Monte Carlo simulation of the CICGN model and (red) circles are those from the PDBRW. The solid lines represent the relations *P*(*n*) ~ *n*^−*α*^ with *α* = 1.50 (**a**), *α* = 1.80 (**b**), *α* = 0.60 (**c**), and *α* = 0.70 (**d**).

As we increase *T*_0_/*N*_0_ further, initial transient behavior of meme popularity distribution is drastically changed. In Fig. 3 we display the measured *P*(*n*)’s for various time intervals with *N*_0_ = 10^4^ and *T*_0_ = 10^3^. As shown in Fig. 3(a,b), measured *P*(*n*)’s both on RN and SFN for *t* = 0 ~ 10 satisfy the power-law, unlike the case of *N*_0_ = 10^4^ and *T*_0_ = 10. From the best fit of the data to Eq. (1), we obtain *α* = 1.5 ± 0.1 on RNs and *α* = 1.8 ± 0.1 for SFNs. The obtained *P*(*n*) only for this special initial condition, in which *T*_0_/*N*_0_ is relatively large, coincides with the suggested behavior of *P*(*n*) in ref. 15. *α* on SFNs deviates from *α* = 3/2 but close to *α* = 2, but we can not exclude the suggested relation *α* = *γ*/(*γ* − 1)^15^,^20^. The results are also confirmed by the numerical analysis of the PDBRW.

For the intermediate time interval, some memes spread over a wide area on the network. On the other hand some memes disappear due to the competition. As a result, the memes spread over a wide area have more chance to be propagated to the connected nodes. Due to the absence of innovation process for *μ* = 0, there is no way to stop the propagation of such dominant memes. Thus, the dominant memes acquire more popularity than the non-dominant memes and the heterogeneity in the popularity increases. Resultantly, *α* decreases as *t* increases (see Fig. 3(c,d)).

From the data in Fig. 3(c,d), we obtain *α* = 0.6 ± 0.1 for RN and *α* = 0.7 ± 0.1 for SFN when *t* = 40 ~ 50. The values of *α* when *t* = 40 ~ 50 both for RN and SFN are nearly same. Finally, as *t* → ∞ there left only one meme on the network when *μ* = 0.

#### CIC model with 0 < *μ*′ < 1

**Figure 4:**
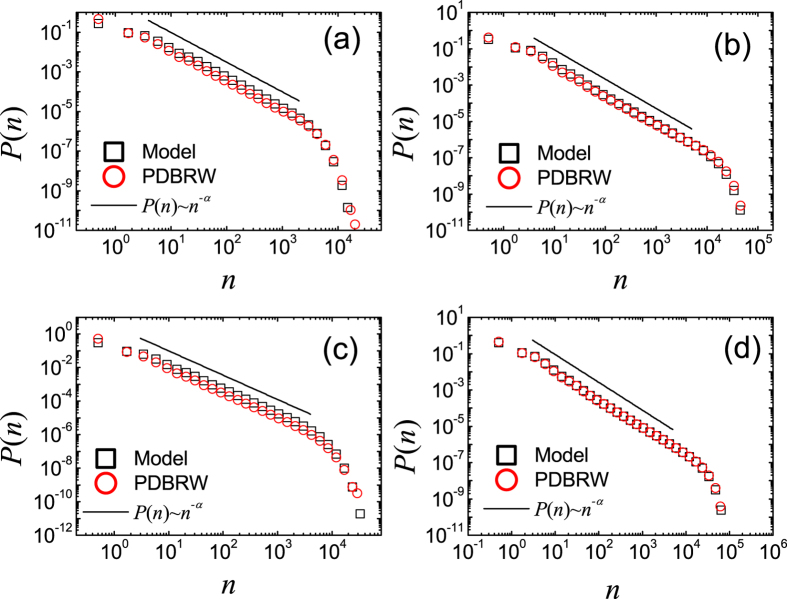
Plots of *P*(*n*) of the CIC model with *μ*′ = 0.01, *T*_0_ = 10^3^ and *N*_0_ = 10^4^ for *t* = 0 ~ 10 on RNs (**a**), for *t* = 0 ~ 10 on SFNs with *γ* = 2.5 (**b**), for *t* = 990 ~ 1000 on RNs (**c**) and for *t* = 990 ~ 1000 on SFNs with *γ* = 2.5 (**d**). (Black) Squares are *P*(*n*) obtained from direct Monte Carlo simulation of the CIC model and (red) circles are those from the PDBRW. The solid lines represent the relations *P*(*n*) ~ *n*^−*α*^ with *α* = 1.50 (**a**), *α* = 1.45 (**b**), *α* = 1.60 (**c**), and *α* = 1.53 (**d**).

In order to investigate how the innovation process affects *P*(*n*), we also investigate the CIC model with *μ*′ > 0. In Fig. 4 we show the measured *P*(*n*)’s from the direct numerical simulation of CIC model and the PDBRW on both networks when *t* = 0 ~ 10 and *t* = 990 ~ 1000. As shown in Fig. 4(a–d), we find that *P*(*n*) always satisfies Eq. (1) with *α* ≃ 3/2, regardless of the underlying topology and the value of *T*_0_/*N*_0_. In ref. 15 it was suggested that *α* for *μ*′ ≠ 0 crucially affected by the structure of the underlying network, for example, if 2 < *γ* < 3 then *α* = *γ*. However, our analysis clearly shows that if there is the innovation process, then *α* ≃ 3/2 on any networks. This result suggests that the system is poised at the criticality when there occurs the balance between the creation of new memes by the innovation process and the extinction of old memes. Thus, the innovation process constantly provides competitions among the memes. If *μ*′ gets larger, the balance between the creation of new memes and the extinction of old memes breaks down. Too much innovations make the popularity of any meme very small as can be seen from *P*(*n*) = *δ*_*n*,1_ for *μ*′ = 1. Thus, the balance for the criticality sustains for relatively small innovation rate. We confirm by numerical simulation that the criticality exists for 0 < *μ*′ < 0.1.

#### CICGN with 0 < *μ* < 1

**Figure 5:**
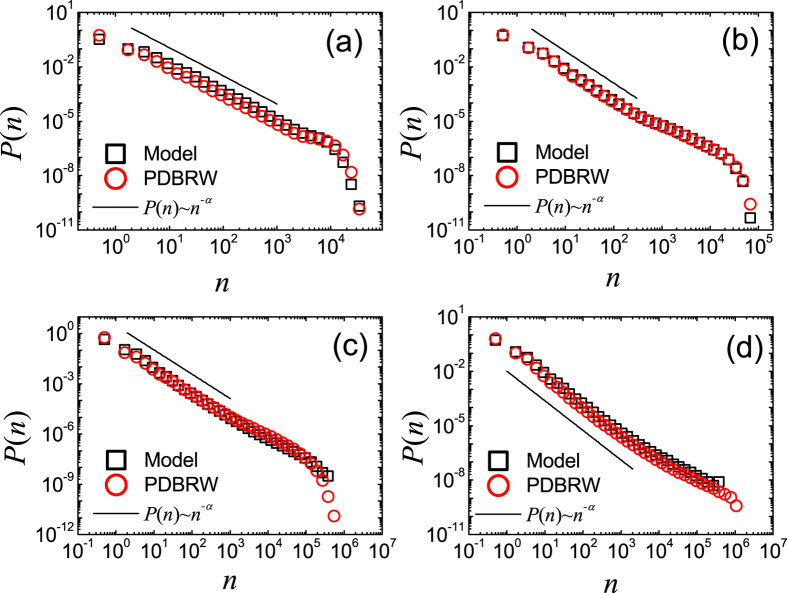
Plots of *P*(*n*) of the CICGN model with *μ* = 100/*N*(*t*), *T*_0_ = 10^3^ and *N*_0_ = 10^4^ for *t* = 0 ~ 10 on RNs (**a**), for *t* = 0 ~ 10 on SFNs with *γ* = 2.5 (**b**), for *t* = 990 ~ 1000 on RNs (**c**) and for *t* = 990 ~ 1000 on SFNs with *γ* = 2.5 (**d**). (Black) Squares are *P*(*n*) obtained from direct Monte Carlo simulation of the CICGN model and (red) circles are those from the PDBRW. The solid lines represent the relations *P*(*n*) ~ *n*^−*α*^ with *α* = 1.5 ± 0.1 (**a**), *α* = 1.6 ± 0.1 (**b**), *α* = 1.5 ± 0.1 (**c**), and *α* = 1.6 ± 0.1 (**d**).

Since most of the real online social networks grow, it is also important to study the effect of the growth on the popularity of meme. By definition of CICGN model, when 0 < *μ* < 1 there is non-zero probability to create a new node with a new meme at each time step. In Fig. 5 we show *P*(*n*)’s during two different time intervals, *t* = 0 ~ 10 and *t* = 990 ~ 1000 both on RN and SFN. From the best fit of the data to Eq. (1) we obtain *α* = 1.5 ± 0.1 for *t* = 0 ~ 10 (Fig. 5(a,c)) and *α* = 1.6 ± 0.1 when *t* = 990 ~ 1000 (Fig. 5(b,d)) on both networks.

The obtained values of *α* on both networks are the same with those for CIC model with 0 < *μ*′ < 0.1 within the estimated errors. This can be clearly understood from the PDBRW. As already addressed, Eqs (4,5) have the same functional form. Furthermore, if *N*(*t*) is large enough, then CIC model with *μ*′ > 0 and CICGN model with *μ* > 0 are described by the same PDBRW. The condition *N*(*t*) ≫ 1 is easily achieved by the growth of the network with the innovation process. Therefore, through the mapping to the PDBRW, the innovation process is proved to be one of the most fundamental mechanisms which make the system be critical with *α* = 3/2. We also confirm the criticality with *α* ≃ 3/2 regardless of values of *T*_0_/*N*_0_ on SFNs with various *γ*’s.

On the growing networks, the balance between the creation of new memes by the innovation process and the extinction of old memes is also the key factor for the criticality with *α* = 3/2. We confirm that the balance for the criticality sustains for relatively small innovation rate or 0 < *μ* < 0.1 in CICGN model. This result means that some memes acquire large popularity through the evolution and become impactable memes in CIC or CICGN model with relatively small innovation rate. In real social networks, the innovations with such impactable memes should not happen very often. Therefore, the innovation processes with significantly influential memes should be the simple and fundamental processes which cause the power-law or heavy-tailed distribution of the meme popularity in various real social networks.

## Discussion

In summary, we study the meme popularity distribution on non-growing and growing networks based on the CIC. From the direct Monte Carlo simulation of the model, we find that the meme popularity distribution, *P*(*n*), is single peaked and highly skewed for initial transient period if *μ*′ = 0 in CIC model and *μ* = 0 in CICGN model when *T*_0_/*N*_0_ is sufficiently small. For intermediate time interval, we find that *P*(*n*) satisfies Eq. (1) with *α* ≃ 0.6 on RN and *α* ≃ 0.8 on SFN, respectively. As we increase the ratio *T*_0_/*N*_0_, we find that the initial transient behavior of *P*(*n*) is drastically changed and satisfies the power-law with *α* ≃ 1.5 on RN and *α* ≃ 1.8 on SFN while the value of *α* for intermediate period is decreased to *α* = 0.6 ~ 0.7 on both networks. However, for small *μ*′(0 < *μ*′ < 0.1) and *μ*(0 < *μ* < 0.1), we obtain *α* ≃ 3/2. This value of *α* is robust and does not depend on the ratio *T*_0_/*N*_0_, the underlying topology, and the measured time interval. It contradicts the suggested behavior of *P*(*n*) in ref. 15. Furthermore, we also map the meme creation and propagation dynamics into the PDBRW. One of practical advantages of the PDBRW is that we can increase *N*(*t*) as large as we want, which enables to obtain more accurate numerical results compared with the direct Monte Carlo simulation of the CIC and CICGN models. In addition, from the exact mapping into PDBRW, we show that evolution of *x*(*t*) in the CIC model with *μ*′ > 0 has the same functional form with that of CICGN model with *μ* > 0. Especially, if *N*(*t*) ≫ 1 then Eqs (4) and (5) become exactly identical.

The effect of the location of innovation is also investigated through the growing network model in which only the old nodes generate the new memes with probability *μ*. In this case, the mapping into PDBRW shows that the effect of growth on the evolution of *x*(*t*) becomes negligible if *N* ≫ 1. Thus the scaling behavior of meme popularity distribution of this model should be the same with that in CIC or CICGN models. It is also verified by the numerical analysis.

The models investigated in our present study does not consider any detailed features such as the fitness of memes, aging, or external events. Due to its simplicity, we clearly show that the most fundamental mechanism which produces the criticality or the power-law distribution of meme is the innovation which makes the balance between the creation of new memes and the extinction of old memes. In real situations, the size of many social networks grows exponentially and the innovations with impactable memes should not happen very often. Therefore, the innovation processes with significantly influential memes should be the simple and fundamental processes which cause the power-law or heavy-tailed distribution of the meme popularity in various real social networks.

Our results do not imply that the detailed features of memes play no role in characterizing the scaling behavior of meme popularity. Since our model is one of the simplest models to account for the origin of the criticality in meme popularity distribution, how the additional features such as fitness of meme and aging affect the scaling behavior of meme popularity might be another important open question to be answered to enhance our understanding of the human behavior in the information world.

## Additional Information

**How to cite this article**: Kim, Y. *et al.* The origin of the criticality in meme popularity distribution on complex networks. *Sci. Rep.* **6**, 23484; doi: 10.1038/srep23484 (2016).
